# Older Adults’ Experiences, Worries and Preventive Measures Regarding Home Hazards: A Survey on Home Safety in Sweden

**DOI:** 10.3390/ijerph20021458

**Published:** 2023-01-13

**Authors:** Elin Mauritzson, Kevin J. McKee, Marie Elf, Johan Borg

**Affiliations:** School of Health and Welfare, Dalarna University, 791 88 Falun, Sweden

**Keywords:** home safety, preventive behaviour, national survey, older adults, +70 years of age, self-reported health, neighbourhood, worry, community living, social support

## Abstract

Home safety is important for preventing injuries and accidents among older adults living at home. Feeling safe at home is also essential for older adults’ well-being. Thus, this study aimed to explore older adults’ perceptions of safety in their homes by examining their experiences, worries and preventive measures in relation to a range of potential home-based health and safety hazards. The study was a national cross-sectional telephone survey of 400 randomly selected adults over 70 years of age living at home in ordinary housing in Sweden. Participants were asked for their experience of, worry about, and preventive measures taken regarding fifteen home hazards. Data were also collected on background variables including age, health, and cohabitation status. Falls and stab/cut injuries were the most experienced hazards and worry was highest for burglary and falls, while preventive measures were most common for fire and burglary. While older adults’ experience and worry regarding home hazards were associated with preventive measures, these associations were not strong and other factors were associated with preventive behaviour. Further identification of the main determinants of older adults’ preventive behaviour can contribute to policy for effectively reducing home accidents.

## 1. Introduction

### 1.1. Importance of the Home

It is estimated that older adults aged 65 years or over make up a fifth of the Swedish population but account for 7 out of 10 fatal accidents and 5 out of 10 people who need care due to accidents [1,2]. These figures correspond to those of other OECD countries [3]. Many of these accidents occur in the older adults’ homes or close neighbourhoods [2]. Older adults often have complex health conditions such as sensory problems, stroke, and dementia that can contribute to impaired functioning and thus they have an increased risk of accidents and injuries [3,4]. Falls are the most common cause of injuries in the home for older adults, but burns and corrosions, poisonings, and cuts or stab wounds are also common [2,5]. Injuries and accidents can, in turn, lead to health problems and functional limitations that result in loss of independence and even institutionalisation [6]. Many older adults with poor health spend most of their time in their homes [7], making it essential that the home is a safe environment. Research that explores older adults’ perceptions and experiences of their home, including feelings of safety, occurrences of accidents and injuries, and preventive measures against home hazards, can contribute to that goal.

The Swedish “kvarboende” residence principle encourages older adults to stay at home for as long as possible [8]. “Aging in place” is a general term for similar policies in many European countries, which focus on enabling older adults to live in their ordinary homes rather than in special housing or institutions [9]. Aging in place can contribute to a sense of identity by supporting independence and autonomy and through caring relationships and meaningful roles in the places where people live. Research has shown that older adults often want to live in their own homes for as long as possible, even when they experience frail health [10,11]. On the other hand, places in special housing in Sweden were reduced by over 30% between 2001 and 2015 [12]. Thus, some older adults may prefer to move to special housing but cannot due to the lack of provision. The aging in place policy and the reduction in special housing have jointly contributed to an increasing number of older adults living in their own homes to more advanced ages, often with the help of home-based care and home adaptations [13].

### 1.2. Safety at Home

Feeling safe at home is essential for older adults’ well-being [2,14]. When an older adult’s home can no longer provide safety and support, vulnerability can increase, and the home can be perceived as a threat [2]. Schröder-Butterfill and Mariantis [15] proposed a model for understanding vulnerability in older adults, in which their vulnerability is seen as the outcome of an interaction of risks: the risk of being exposed to a threat, the risk of a threat materialising, and the risk of lacking the defences or resources to deal with a threat.

The experience of feeling unsafe in the home can lead to stress, lower self-efficacy, a perceived loss of control and lower health-related quality of life for the older adult [16]. While research on safety at home for older adults often focuses on accident and injury prevalence [17], safety at home is more than just the absence of accidents. It is also about a feeling of at-homeness in the neighbourhood, being able to maintain independence and, for those in receipt of home care, being able to influence the service and trust staff [18]. When older adults can make their own decisions about home care services or social interaction, this can contribute to them feeling safe.

Different groups of researchers have conceptualised home safety in different ways. One conceptualisation is that there are four components to safety at home for older adults: living in a familiar place; having few and manageable fears; obtaining services when needed; and learning and adapting knowledge about home safety [11]. Another group of researchers have proposed four dimensions of home safety: physical (for example, medication, home improvement, falls related to failing health and use of technology), social (relationships on their own terms with other people), emotional and mental (having a secure feeling at home and trustful relationships with homecare staff), and cognitive (issues relating to declining cognitive functioning) [11]. Other work has placed older adults’ perceptions of safety in three contexts: the home environment; the outdoor environment and traffic; and the digital environment, and differentiates between perceptions of safety that are impacted by intentional acts and negligence (for example, burglary and violence) and those impacted by non-intentional acts (for example, accidents, making mistakes online) [19]. In our study, we chose to operationalise safety in relation to older adults’ own perceptions of three key aspects of their home environment: experience, worry, and prevention.

Older adults who take preventive measures towards hazards in the home can often stay at home for longer [20]. Preventive measures in the home can improve more than one safety dimension, including improved quality of life, independence in daily living, and reduced fear of falling [21]. Applying Schröder-Butterfill and Mariantis’s [15] vulnerability framework described above, various strategies can be proposed to reduce an older adult’s feelings of vulnerability In the home via preventive measures. Taking the threat of water damage in a room as an example, this can be reduced by shutting off the water supply to the room (reduces the risk of being exposed to the threat), using high-quality fittings, pipes, and mixers that are correctly mounted (reduces the risk that the threat is realised), and using dense surface layers and a drain in the room (protects against a realised threat).

Preventive measures in the home can include kitchen modifications, home lighting, and small-scale modifications such as installing bath grab rails [21]. They can also involve more advanced technology, for example, various assistive products for daily life that include digital technology, such as security alarms, surveillance via camera, sensors for reminders, robots, and computer programs [22]. Technology for ageing at home has been developed over many years and often focuses on a healthy lifestyle, loneliness, safety and distance care [23].

Many older adults accept the need for preventive measures, while others do not, which can hinder safety at home [24]. While there is relatively little research on why a person takes preventive measures towards hazards in their home, such behaviour can be placed in the context of social cognition models that have been successfully used to predict a wide spectrum of preventive health behaviours and health behaviour change [25]. A key general theory that underpins several social cognition models is expectancy–value theory, in which it is argued that people are motivated to perform a task if they believe they have the ability (expectancy) and will benefit directly or indirectly (value) from doing so [26]. In the context of home safety, expectancy-value theory would predict that an older adult will be highly motivated to take preventive measures if they perceive themself at risk of experiencing a particular hazard that would have severe consequences, while possible preventive actions would be evaluated in terms of their potential personal benefits and costs. For example, older adults can choose not to take preventive measures because of financial restrictions or thriftiness [27]. If older adults own their house or not can also affect the availability of preventive measures and their perceived safety; for example, older adults who own their home report fewer hazards such as burglary [28]. Many older adults participate in prevention programmes, for example for falls, which can raise awareness and intention to take preventive measures [29,30]. However, there are also examples of when prevention programmes can result in older adults perceiving their home as less safe [31].

### 1.3. Aim and Research Questions

Research on safety in the homes of older adults has often focused on a specific issue or group, for example fall accidents or persons with dementia, and mainly explored the carer’s perspective. There is a need for more knowledge about safety in the homes of older adults based on their own perceptions and experiences. Such knowledge is necessary to guide effective interventions to increase feelings of safety among older adults. Thus, this study aimed to explore older adults’ perceptions of safety in their homes by examining their experiences, worries, and preventive measures in relation to a range of potential home-based hazards. The research questions were:

1. What home hazards have older adults experienced and taken measures to prevent?
2. How worried are older adults about home safety and specific home hazards?
3. To what extent are older adults’ worries about, experiences of, and preventive measures towards home hazards related to each other?
4. How are demographic, psychosocial, and health factors related to older adults’ experiences of, worries about, and preventive measures towards home hazards?

## 2. Materials and Methods

### 2.1. Design

The study was a national cross-sectional questionnaire-based telephone survey of older adults living at home in Sweden.

### 2.2. Sampling and Participants

Persons were eligible for the study if they were aged 70 years or older, lived in Sweden in ordinary housing, and spoke Swedish. The age group was chosen in order to include adults that have retired. Although the official retirement age in Sweden is 65 years, it is not uncommon to work until 69 years of age. Potential participants were randomly selected from a database created for this study based on the contact details of subscriber data from databases of all phone operators in Sweden. Only individuals whose identities could be verified through official data from the Swedish Tax Agency were included in the database.

The sample size calculation was based on the intention to estimate the prevalence of experiences of hazards and the prevalence of taking measures to prevent hazards in the Swedish population aged 70 and older within 10% of the true prevalence with a confidence level of 95% [32]. As the prevalence was unknown, it was assumed to be 50%, resulting in the largest required sample size, i.e., 384 [33]. Given that there might be some non-response, a sample size *n* = 400 was targeted in this survey.

Sampling from the database continued until the desired sample size was attained. In this process, attempts were made to contact 2824 people, of whom 1466 could not be contacted or were found to be ineligible upon contact. Of the remaining 1358 people who were contacted and eligible, 784 declined (57.7%), 134 interviews were discontinued due to health/language issues (9.9%), 40 interviews were only partially completed (2.9%), and 400 people participated and provided complete interviews, corresponding to a response rate of 29.5%.

### 2.3. Materials

A questionnaire was developed according to an established process for questionnaire development [34]. Development was in three phases: (1) design (item generation, design of the survey); (2) revision (cognitive interviews with experts); and (3) adaptation (pilot testing and audit). In the design phase, the development was guided by (a) core concepts from a theoretical framework on vulnerability in older adults [15] and expectancy–value theory, (b) a literature review on perceptions and experiences of safety at home among older persons, (c) a random selection of injury claims (*n* = 163) from the Swedish insurance company Länsförsäkringar, (d) existing questionnaires covering similar issues [35,36], and (e) home hazards among older people in Sweden [2,35]. To avoid lengthy interviews, a preliminary selection of the most common and severe hazards was limited to fifteen in number. The first pool of items was reviewed and revised in an iterative process. In the revision phase, ten experts on community-living older adults were recruited through the research team’s networks and asked to comment on and rate the questionnaire items regarding their relevance and comprehensibility. These ratings were used to assess the questionnaire’s content validity by calculating a content validity index (CVI) [37]. Three questions with a lower CVI score (under 0.90) for comprehensibility were revised. In the adaptation phase, the questionnaire was pilot tested among two individuals recruited through the research team’s networks who met the eligibility criteria for study participants [34]. They were interviewed by phone using the developed questionnaire and asked to comment and rate the questions’ relevance and comprehensibility. This did not lead to any further revision of the items.

The final questionnaire consisted of 57 questions (see Supplementary Materials): 9 demographic and housing-related questions; 3 questions about the importance of feeling safe in the home; and a question on each of experiences, worries about, and preventive measures toward 15 different home hazards (thus, 45 separate questions).

### 2.4. Procedure

The survey was conducted in September 2021 by professional interviewers from a data collection and management agency. Sampled individuals were sent written information about the purpose of the study, how they had been identified as potential participants, and confirmation that participation in the study was voluntary. Approximately one week after receiving this information, these individuals were telephoned by the interviewers who provided further details of the survey, answered any questions, and sought informed consent. On consent, the interview began. The interviewers could discontinue the interviews on their discretion or if the interviewee preferred to do so. The median interview time was 7 min 26 s. The study was approved by the Swedish Ethical Review Authority (reg.no. 2021-01706).

### 2.5. Data Analysis

IBM SPSS v. 28 was used for all data analysis. Descriptive statistics were produced for participants’ characteristics and responses to the questionnaire items, means and standard deviations for interval and scaled items, and number and percentages for discrete and dichotomous items. Bivariate associations between items were analysed as follows: Spearman’s rho was calculated for associations between interval and scaled items and associations between dichotomous and interval/scaled items; the phi coefficient was calculated for associations between dichotomous variables. As per convention, a *p*-value below 0.05 was considered statistically significant. No adjustment was made for multiple testing, and so the significance of each test result should be considered in the context of the obtained effect size.

## 3. Results

### 3.1. Characteristics of the Participants

Table 1 presents participants’ demographic and background characteristics. The sample was gender-balanced and participants had a mean age of 77.6 years. Nearly three-quarters of the participants owned their house or apartment, while a majority co-habited. Compared with co-habiting participants, higher proportions of those who lived alone lived in rented accommodations and in apartments. Relatively few participants received home care, required support for indoor mobility, or had experienced financial difficulties in the previous 12 months. The average participant rated the frequency of receiving support when needed as often; their general health as good; feeling unsafe at home as seldom; feeling safe at home as very important; and feeling of safety in their neighbourhood as quite or very safe.

### 3.2. Experiences of, Worries about, and Preventive Measures towards Hazards

Table 2 shows that the prevalence of experiencing home hazards ranged from 2.0% to 24.5% across 15 hazards. The mean level of worry across the 15 hazards ranged from M = 1.58 to M = 2.53, while the prevalence of preventive measures ranged from 11.1% to 85.0%. The table also presents the sample-level rank order of the hazards by prevalence of experience, mean level of worry, and prevalence of preventive measures. Fall or slip accident was the most commonly experienced hazard, worry was highest for burglary, and fire was the hazard that most participants had taken preventive measures towards.

**Figure 1:**
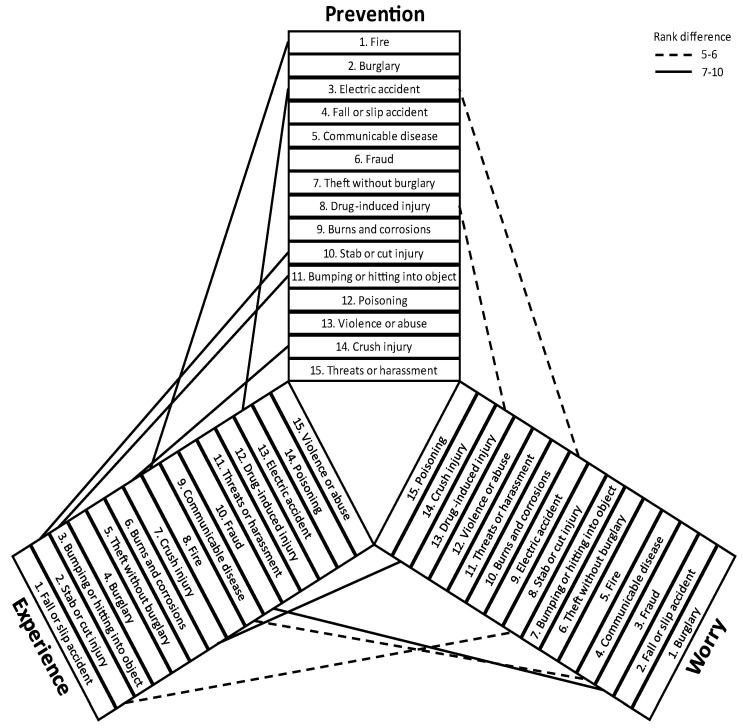
Illustration of the relationships between the sample-level rank order of 15 hazards by prevalence of experience, prevalence of prevention and mean level of worry.

Figure 1 illustrates relationships between the sample-level rank order of the hazards by prevalence of experience, prevalence of prevention, and mean level of worry. The overall pattern is for the largest discrepancies in the hazard rank orders to be between those for experience and prevention, with the smallest discrepancies between worry and prevention, and a moderate level of discrepancies between worry and experience. Stab or cut injury and impact injury had high rankings in experience but lower rankings in worry and preventive measures. Crush injuries had a high ranking in experience, but a low ranking in prevention. The hazards fire and electrical accidents had low rankings in experience but higher rankings in worry and prevention. Fraud had low rankings in experience and preventive measures but a higher rank in worry. The hazards burglary and fall or slip accident ranked high on all three safety aspects.

The associations between experience, worry, and preventive measures for all hazards are shown in Table 3. Most bivariate associations between hazard experience and preventive measures were significant, with the coefficients ranging in size from 0.11 for burn or corrosion injury to 0.27 for both infectious disease and drug-induced injury. The associations were non-significant for four hazards: fire, electrical accident, poisoning and crush injury. Most bivariate associations between hazard experience and worry were significant, with the coefficients ranging in size from 0.05 for poisoning to 0.29 for stab or cut injury. The associations were non-significant for four hazards: fire, electrical accident, poisoning and violence or abuse. Most bivariate associations between hazard worry and preventive measures were also significant, with the coefficients ranging in size from 0.11 for burn or corrosion injury and 0.30 for burglary. The associations were non-significant for two hazards: fire and crush injury.

### 3.3. Demographic- and Housing-Related Factors Associated with Prevalence of Experience, Worry about, and Preventive Measures towards Hazards

Table 4 shows the associations between selected demographic and background variables and participants’ experiences of each hazard. Considering the significant positive associations for all demographic and background variables, higher age was associated with experience of a fall or slip accident. Feeling more unsafe at home was associated with experience of 7 of the 15 hazards (range *r_s_* = 0.11–0.26). Living alone was associated with experience of fraud. Higher frequency of receiving support when needed was associated with experience of crush injury and stab or cut injury. Considering the significant negative associations, higher age was associated with no experience of fire and threat or harassment. Feeling more safe in the neighbourhood was associated with no experience of threat or harassment and violence or abuse. Living alone was associated with no experience of burglary. Better general health was associated with no experience of fall or slip injury, drug induced injury, and fraud. Female gender was associated with no experience of an electrical accident (φ = −0.14; not shown in table). Importance of safety at home had no significant associations with experience of any hazard.

Table 4 also shows the association by hazard between selected demographic and background variables and participants’ level of worry. Considering the significant associations for all demographic and background variables, higher age was associated with more worry about falls and impact injury. Higher frequency of feeling unsafe at home was associated with more worry about all hazards (range *r_s_* = 0.10–0.31); higher frequency of feeling safe in the neighbourhood was associated with less worry about all hazards (range *r_s_* = −0.12–−0.23) except fire and fraud. Higher frequency of accessing support when needed (not shown in table) was associated with less worry about burglary (*r_s_* = −0.12) and violence/abuse (*r_s_* = −0.10); while better general health was associated with less worry about all hazards (range *r_s_* =−0.11–−0.25) except fire. Gender, importance of safety at home, and living alone had no significant associations with level of worry about any hazard.

Table 4 also shows the association by hazard between selected demographic and background variables and preventive measures taken by participants. Considering the significant positive associations for all demographic and background variables, higher age was associated with preventive measures against falls. Higher frequency of feeling unsafe at home was associated with preventive measures against drug-induced injury, burglary, theft, fraud, and violence/abuse; having better general health was associated with taking preventive measures against fire and electrical accidents, while higher frequency of receiving support when needed was associated with taking preventive measures against electrical accidents (*r_s_* = 0.11; not shown in table) and greater importance of home safety was associated with preventive measures against fraud (*r_s_* = 0.10; not shown in table). Considering the significant negative associations, higher age was associated with not taking preventive measures against fire and electrical accidents. Living alone was associated with not taking preventive measures for nine out of the fifteen hazards (range φ = −0.10–−0.20). Having better general health was associated with not taking preventive measures against falls. Gender and feeling safe in the neighbourhood had no significant associations with taking preventive measures towards any hazard.

## 4. Discussion

### 4.1. Summary of the Findings

This study aimed to explore older adults’ perceptions of safety in their homes, focusing on their experiences, worries, and preventive measures in relation to a range of home-based safety hazards. None of the fifteen hazards had been experienced by more than a quarter of the participants, with the majority experienced by less than one in ten. Preventive measures had been taken by more than a third of the participants for only six of the studied hazards. While participants on average saw safety in the home as important, they had low levels of worry about their safety in the home and in their neighbourhood. Their levels of worry were relatively low for all hazards, being “very little” to “slightly” worried for seven, with worry highest for falls and burglary. Most of the associations examined between participants’ experience, worry, and preventive measures for the studied hazards were significant, albeit all were weak to moderate in strength. Fire was the only hazard for which there were no associations between experience, worry, and prevention. Some background and demographic characteristics were consistently associated with participants’ perceptions of home hazards. Most notably, these included feelings of safety in the home and in the neighbourhood, but also self-reported health, age, and, particularly for preventive measures, cohabitation status.

### 4.2. Perceptions of Safety

The prevalence for experiencing hazards in our sample followed a pattern comparable to that found in the available statistics for hazards experienced by Swedish older adults [2]. Comparative international studies on the experience of home hazards are very rare, with most research conducted on a national or regional level. While both theory and some empirical findings suggest that experiencing a hazard is related to higher levels of worry about that hazard, other studies have produced findings similar to ours, i.e., that older adults can be worried about certain hazards without ever having experienced them [35]. This suggests that older adult’s worry about home hazards is at least partly influenced by other factors than their direct experience, for example if there is a lot of media coverage concerning that hazard. This is one potential explanation for why infectious disease and fraud were respectively the ninth and tenth-ranked hazards for experience, but the fourth and third-ranked hazards for worry. Another possible explanation for the low level of association between experience and worry is that the experience of some hazards can reduce worry about them, either because the experience is not as negative as was anticipated, or because the experience initiates coping responses, such as acquiring and deploying preventive measures.

We found several factors to be associated with participants’ experiences of and worries about hazards, and some of these have also been found to be important in other studies. For example, feeling at home in the immediate neighbourhood [18] and health status [16] have been found to be associated with feelings of safety at home for older adults. Many older adults want to continue living in their own homes as they age [11,38], and the sense of familiarity associated with living in a dwelling and environment for a long time is likely linked to feeling safer [39]. Research has also shown an association between access to and quality of support and feelings of safety [18,38], which is reflected in our study where the frequency of receiving support when needed was associated with the experience of and worry about some hazards.

### 4.3. Preventive Measures

Some social cognition models [25] predict that an older adult’s worry about a hazard should be associated with taking preventive measures against that hazard in their home. Our findings indicate that while experience and level of worry are associated with preventive measures, other factors are also important, including feelings of safety at home, age, health, and cohabitation status. The complexity of hazard-preventing behaviour is also confirmed by other studies, which have found that a variety of factors are potentially involved [24,27,28,29,30,40], such as the awareness and acceptance of the need for prevention, the availability of preventive measures, perception of potential benefits (some measures prevent exposure and some minimise damages), and the ability to take measures due to, e.g., age, financial situation, or ownership of one’s home.

A further reason for the lack of association between experience, worry, and preventive measures could be normative pressure to take preventive action with respect to particular hazards. Where preventive measures are also relatively low cost and available, this may result in most people taking such measures for, e.g., fire and burglary. As such, the lack of variation in prevention of these hazards would restrict the potential for co-variation with experience and worry. One study [41] found that for adults in general, having experienced fire in the home or having an interest in fire prevention did indeed increase the probability of taking preventive measures against fire, while another study [42] found that the determinants of older adults’ preventive behaviour against fire are risk perception, self-efficacy, habits, and perceived barriers such as physical disabilities. The study also found that older adults perceive the risk of home fires as low and are confident in their abilities to act in case of a fire.

Preventive measures in the home have been shown to result in improvements in quality of life and independence in daily living as well as reduced fear of falling [21]. However, there are also examples of where making the issue of hazards and their prevention salient can result in older adults perceiving their home as less safe [31]. There are challenges when adapting an older person’s own home in order to make it a safer environment. Preventive measures vary in their acceptability to and usability by older adults, while introducing a preventive measure into the home environment can have the unintended effect of making it feel less “homelike” to the occupant. Advances in technology have the potential to make preventive measures less invasive and more effective and, through the use of co-design strategies, more acceptable and usable among older adults, although more research is needed [43].

Older adults living alone are an especially important group to consider in prevention initiatives, as our results suggest that they are less likely to take preventive measures than co-habitant older adults, perhaps partly due to lower motivation to act only for oneself. There could also be differences in the types of living accommodation commonly used by these two groups, for example, if those living alone are more likely to live in flats or rented accommodation where there could be fewer possibilities to make alterations, or where landlords have the responsibility for taking preventive measures. One study found that living alone and feelings of safety were associated, but living alone was not always seen as a problem by the older person if they considered everything else in their home environment to be adequate [44].

Taken together, our findings reinforce how important it is that policies intended to increase home safety and the uptake of preventive measures need to be targeted and take account of the heterogeneity in the older population and their living conditions. To better understand the causal connections between older adults’ experiences, worry, and preventive measures relating to home hazards, longitudinal studies are required. Future studies need to target specific groups of older adults that are under-represented in research, for example those in poorer health, in reduced social circumstances, or of minority ethnicity, while in-depth studies using qualitative strategies are needed to provide a richer, more detailed understanding of older adults’ thoughts and feelings on home safety.

### 4.4. Strengths and Limitations

A major strength of this study is its sampling frame. By using national databases, participants were recruited nationally, providing a sample that was broadly representative in terms of the geographical distribution of the Swedish population. The response rate was 29.5%, which is similar to other telephone-based surveys [45], and our sample largely corresponds to what would be expected for older adults within this age range in Sweden on most of the demographic and background characteristics assessed [1,36]. The exceptions were that our sample was healthier and had a lower level of home care use compared to the population [1,2]. This could be because frailer older adults did not want to participate or were not able to participate because of health, cognitive impairment, or language difficulties. Postal and online surveys often have a poor response from frail older adults [46], hence our use of telephone interviews. However, those older adults highly concerned about the risk of fraud or with high levels of general anxiety might have been suspicious of the survey and chose not to participate, despite being informed of the study in advance and our use of experienced professional interviewers. Another strength of this study is that it explores home safety from older adults’ own perspective in relation to a broad range of home hazards. Other studies of home safety have tended to focus on one type of hazard and use family or formal carers as the main source of data [11].

The choice of a cross-sectional study design has limitations, as we cannot infer causal relationships between experience, worry, and preventive measures nor establish a sequence of events, e.g., we do not know when the older adult had an experience of a hazard and if that experience preceded or followed taking preventive measures. Our finding that some of the hazards were experienced by few participants while worry about most hazards was positively skewed may partly explain the relatively weak associations between experiences, worry, and preventive measures. It should be noted that our rankings of hazards by level of experience, worry, and prevention were at the group level. We did not ask participants to rank the hazards, and such individual-level ranking might have produced different results. Finally, the COVID-19 pandemic was ongoing during data collection, and this might also have influenced our results in ways difficult to determine.

## 5. Conclusions

For a range of home hazards, taking preventive measures is consistently associated with older adults’ experience of and worry about the hazards. However, while consistent, these associations are not strong. Other factors, such as age, feelings of safety, access to support, health and cohabitation status, are all related to taking preventive measures against specific hazards in the home. Thus, the adoption of, for example, technological solutions for home hazards by older adults will likely be influenced by many factors. Policymakers and developers of new preventive measures will need to maintain a broad perspective if their efforts are to be effective and ensure that policies and preventive measures are appropriately targeted, acceptable to the user, and user-friendly. The use of co-design strategies when adapting home environments to make them safer when developing solutions for home hazards is recommended.

## Figures and Tables

**Table 1:** Participants’ background characteristics, *n* = 400.

Characteristic	
Age	Mean 77.6 (SD = 5.16; range 70–99)
Gender ^a^	Male 50.7%
Accommodation	Own house 57.5% Rented house 1.5% Own apartment 16.0% Rented apartment 24.5% Senior housing 0.5%
Living alone ^a^	Yes 39.8%
Home care ^a^	Yes 7.0%
Mobility indoors	No 0% Yes, with support 6.0% Yes, independently 94.0%
Financial difficulties ^a^	Yes 5.3%
Frequency of receiving support when needed 1 (never)–5 (always)	Mean 4.29 (SD = 1.00)
General health1 (very poor)–5 (very good)	Mean 4.16 (SD = 0.79)
Unsafe at home 1 (never)–5 (always)	Mean 1.43 (SD = 0.73)
Importance of safety at home 1 (not at all important)–5 (essential)	Mean 4.10 (SD = 0.68)
Unsafe or safe in neighbourhood 1 (very unsafe)–4 (very safe)	Mean 3.53 (SD = 0.58)

Note: ^a^ For dichotomous variables, response percentage is shown for only one category. Due to non-response, living alone *n* = 399; economic difficulties *n* = 399; frequency of receiving support when needed *n* = 397; general health *n* = 399; unsafe at home *n* = 399; safe or unsafe in neighbourhood *n* = 399.

**Table 2:** Participants’ experience of, worry about, and preventive measures taken towards 15 home hazards.

Hazard	Experience Yes %	Rank ^a^	Worry ^b^ M (SD)	Rank^a^	Prevention Yes %	Rank ^a^
Fall or slip accident	24.5%	1	2.48 (1.18)	2	42.9%	4
Stab or cut injury	22.3%	2	1.93 (1.01)	8	17.8%	10
Impact injury	17.0%	3	2.00 (1.06)	7	17.1%	11
Burglary	16.3%	4	2.53 (1.25)	1	61.8%	2
Theft without burglary	12.8%	5	2.16 (1.18)	6	33.1%	7
Burns and corrosions	11.8%	6	1.86 (1.02)	10	21.6%	9
Crush injury	11.3%	7	1.69 (0.91)	14	11.6%	14
Fire	9.8%	8	2.24 (1.19)	5	85.0%	1
Infectious diseases	9.5%	9	2.32 (1.35)	4	42.4%	5
Fraud	8.5%	10	2.39 (1.30)	3	33.3%	6
Threats or harassment	7.0%	11	1.85 (1.13)	11	11.1%	15
Drug-induced injury	6.0%	12	1.75 (1.01)	13	24.6%	8
Electrical accident	3.0%	13	1.87 (1.14)	9	50.9%	3
Poisoning	2.0%	14	1.58 (1.03)	15	14.4%	12
Violence or abuse	2.0%	15	1.79 (1.16)	12	13.4%	13

Note: ^a^ Rank refers to the rank order of hazards at the sample level in terms of the prevalence of experience, the prevalence of preventive measures, and the mean level of worry. ^b^ Measured on a 5-point scale: 1 = not at all worried, 5 = very worried. For experience of hazards, due to non-response *n* ranges from 398 to 400; for worry about hazards *n* ranges from 398 to 400; for prevention of hazards *n* ranges from 395 to 400.

**Table 3:** Associations between experience, worry and preventive measures for 15 hazards.

Association:	Experience and Preventive Measures (φ)	Experience and Worry (r_s_)	Worry and Preventive Measures (r_s_)
Hazard			
Fire	0.09	0.06	0.07
Electrical accident	0.03	0.07	0.13 *
Fall or slip	0.21 **	0.28 **	0.16 **
Impact injury	0.15 **	0.21 **	0.18 **
Crush injury	0.09	0.18 **	0.07
Stab or cut injury	0.16 **	0.29 **	0.13 *
Burn or corrosion injury	0.11 *	0.19 **	0.11 *
Infectious disease	0.27 **	0.20 **	0.29 **
Drug-induced injury	0.27 **	0.16 **	0.14 **
Burglary	0.24 **	0.24 **	0.30 **
Theft without burglary	0.21 **	0.18 **	0.29 **
Fraud	0.24 **	0.28 **	0.28 **
Threat or harassment	0.19 **	0.28 **	0.17 **
Violence or abuse	0.21 **	0.08	0.17 **
Poisoning	0.09	0.05	0.14 **

Note: *: *p* < 0.05; **: *p* < 0.01. Due to non-response, *n* for associations between experience and preventive measures ranges from 394 to 400; for associations between experience and worry from 396 to 400; for worry and preventive measures from 394 to 400.

**Table 4:** Associations between demographic and background variables with experience, worry, and preventive measures for 15 hazards.

Hazard		Age	Unsafe at Home ^a^	Unsafe or Safe in Neighbourhood ^b^	Living Alone	General Health ^c^
*Experience*	Fire	−0.12 *	0.06	−0.01	−0.02	−0.01
	Electrical accident	0	−0.06	0.1	−0.02	0.02
	Fall or slip	0.14 **	0.12 *	−0.02	0.03	−0.16 **
	Impact injury	−0.02	0.10 *	−0.05	−0.08	−0.03
	Crush injury	0.02	0.14 **	−0.08	−0.02	0.02
	Stab or cut injury	−0.08	0.08	0.07	−0.04	0.05
	Burn or corrosion injury	−0.09	0.04	−0.06	−0.04	0.03
	Infectious disease	0.06	0.008	0.05	−0.09	−0.09
	Poisoning	0.09	0.02	−0.02	0.07	−0.06
	Drug induced injury	0.08	0.07	−0.03	0.08	−0.15 **
	Burglary	−0.06	0.14 **	−0.05	−0.11 *	−0.08
	Theft without burglary	−0.004	0.07	−0.04	−0.05	−0.07
	Fraud	0.02	0.11 *	−0.07	0.10 *	−0.13 *
	Threat or harassment	−0.11 *	0.26 **	−0.11 *	0.04	−0.04
	Violence or abuse	0.01	0.12 *	−0.10 *	0.03	−0.02
*Worry*	Fire	−0.02	0.12 *	−0.10	−0.04	−0.04
	Electrical accident	0.04	0.15 **	−0.13 *	−0.01	−0.11 *
	Fall or slip	0.15 **	0.21 **	−0.14 **	0.05	−0.25 **
	Impact injury	0.12 *	0.16 **	−0.20 **	−0.04	−0.21 **
	Crush injury	0.08	0.18 **	−0.13 *	0.004	−0.15 **
	Stab or cut injury	0.1	0.14 **	−0.14 **	0.03	−0.16 **
	Burn or corrosion injury	0.1	0.14**	−0.19**	0.006	−0.19**
	Infectious disease	0.06	0.14 **	−0.13 *	0.01	−0.15 **
	Poisoning	0.05	0.10 *	−0.12 *	0.04	−0.15 **
	Drug induced injury	0.07	0.17 **	−0.14 **	0.04	−0.23 **
	Burglary	−0.02	0.24 **	−0.23 **	−0.06	−0.16 **
	Theft without burglary	0.01	0.20 **	−0.15 **	−0.10	−0.13 **
	Fraud	−0.05	0.15 **	−0.10	−0.07	−0.12 *
	Threat or harassment	−0.002	0.30 **	−0.21 **	−0.01	−0.12 *
	Violence or abuse	0.04	0.25 **	−0.20 **	−0.03	−0.13 *
*Preventive measures*	Fire	−0.13 *	0.07	0.04	−0.12 *	0.10 *
	Electrical accident	−0.16 **	0.03	0.05	−0.20 **	0.11 *
	Fall or slip	0.16 **	0.02	0.04	−0.05	−0.12 *
	Impact injury	0.08	−0.04	0.05	−0.12 *	0.004
	Crush injury	0.06	−0.002	0.04	−0.10 *	−0.01
	Stab or cut injury	0.03	0.03	0.03	−0.04	−0.04
	Burn or corrosion injury	0.05	0.06	−0.02	−0.11 *	−0.03
	Infectious disease	0.06	0.07	−0.04	−0.07	−0.03
	Poisoning	−0.09	0	0.02	−0.13 *	0.07
	Drug induced injury	0.01	0.11 *	0.01	−0.05	−0.06
	Burglary	−0.05	0.14 **	−0.03	−0.14 **	0.01
	Theft without burglary	−0.03	0.18 **	0.01	−0.18 **	0.01
	Fraud	−0.11 *	0.13 *	−0.03	−0.14 **	−0.06
	Threat or harassment	0.08	0.06	−0.06	−0.09	0.02
	Violence or abuse	−0.003	0.12 *	−0.07	−0.03	−0.01

*: *p* < 0.05; **: *p* < 0.01; ^a^ measured on a 5-point scale: 1 = never, 5 = always; ^b^ measured on a 4-point scale: 1 = very unsafe, 4 = very safe; ^c^ measured on a 5-point scale: 1 = very poor, 5 = very good.

## Data Availability

The data presented in this study are available on request from the corresponding author. The data are not publicly available due to restrictions in accordance with the Swedish Public Access to Information and Secrecy Act (2009:400).

## Supplementary Materials

The following supporting information can be downloaded at: https://www.mdpi.com/article/10.3390/ijerph20021458/s1, Questionnaire.
