# Supplementary material for: Older Adults’ Experiences, Worries and Preventive Measures Regarding Home Hazards: A Survey on Home Safety in Sweden
Source: Int J Environ Res Public Health. 2023 Jan 13;20(2):1458. doi: 10.3390/ijerph20021458 (PMC9859075; doi:10.3390/ijerph20021458)
Supplement: Supplementary file 1 [file ijerph-20-01458-s001.zip › ijerph-2075345-supplementary.pdf]

Supplementary materials
Questionnaire

No.	Question text	Response values and response options [skipping]
I1	Date of interview	
I2	Interviewer ID	
I3	Respondent's phone number	
I4	Based on what I have told you, do you want to answer the questions in the survey?<br><br>*Instruction: Do not read the answer options.*	0 = NO<br>1 = YES [go to Q1]
I5	Do you want to say why you do not want to answer the questions?<br><br>*Instruction: Do not read the answer options. End the interview after this question.*	1 = Memory problem<br>2 = Language<br>3 = Hearing loss<br>4 = Not a good time<br>5 = Do not have time<br>6 = Not interested<br>7 = Do not like to talk on the phone<br>8 = Feeling uncomfortable / insecure<br>9 = No reason<br>10 = Other<br>88 = No response<br>[end the interview after this question]
Q1	How old are you?<br><br>*Instruction: End the interview if the respondent is 69 years or younger.*	[2-digit number]<br>[if 69 or less, end the interview]
Q2	What type of housing do you live in?<br><br>*Instruction: Do not read the answer options initially. If necessary, ask for clarification among the response options that may be relevant.*<br><br>*End the interview if the respondent answers 'Special housing/care facility".*	1 = Own house (terraced, semi-detached or detached<br>2 = Rented house (terraced, semi-detached or detached<br>3 = Condominium in multi-apartment building<br>4 = Rented apartment in multi-apartment building<br>5 = Sheltered housing / senior housing<br>6 = Special housing/care facility [end the interview]<br>88 = Non answer (Do not read)
Q3	How important is it for you to feel safe in your own home?	5 = Essential<br>4 = Very important<br>3 = Quite important<br>2 = Slightly important<br>1 = Not important at all<br>88 = No answer (Do not read)
Q4	How often do you feel unsafe when you are at home?	5 = Always<br>4 = Often<br>3 = Sometimes<br>2 = Rarely<br>1 = Never<br>88 = No answer (Do not read)
Q5	I will now read out a number of events and ask you to state for each event how worried you are to be affected by that event in your home, on a scale of 1-5 where 1 means Not at all worried and 5 means Very worried.<br><br>*Instruction: If necessary, remind about the scale.*	[scale]<br>1 = Not at all worried<br>2 = Very slightly worried<br>3 = Slightly worried<br>4 =Quite worried<br>5 = Very worried<br>88 = No answer (Do not read)<br><br>[rows]<br>Fire<br>Electric accident

		Fall or slip accident<br>Impact injury<br>Crush injury<br>Stab or cut injury<br>Burn or corrosion<br>Infectious disease<br>Poisoning<br>Drug-induced injury<br>Burglary<br>Theft in the home without burglary<br>Fraud<br>Threats or harassment<br>Violence or abuse
Q6	I will now read out the same events again and ask you to answer yes or no to the question of whether you or anyone else has taken any precautionary measures to prevent that event in your home.<br><br>*Instruction: Do not read the answer options.*	[scale]<br>0 = No.<br>1 = Yes<br>88 = No answer (Do not read)<br><br>[rows]<br>Fire<br>Electric accident<br>Fall or slip accident<br>Impact injury<br>Crush injury<br>Stab or cut injury<br>Burn or corrosion<br>Infectious disease<br>Poisoning<br>Drug-induced injury<br>Burglary<br>Theft in the home without burglary<br>Fraud<br>Threats or harassment<br>Violence or abuse
Q7	I will now read out the same events again and ask you to answer yes or no to the question of whether you have ever experienced such an event in your home that made you worried about your safety or health.<br><br>*Instruction: Do not read the answer options.*	[scale]<br>0 = No.<br>1 = Yes<br>88 = No answer (Do not read)<br><br>[rows]<br>Fire<br>Electric accident<br>Fall or slip accident<br>Impact injury<br>Crush injury<br>Stab or cut injury<br>Burn or corrosion<br>Infectious disease<br>Poisoning<br>Drug-induced injury<br>Burglary<br>Theft in the home without burglary<br>Fraud<br>Threats or harassment<br>Violence or abuse
Q8	How safe or unsafe do you feel outdoors in your neighbourhood?	4 = Very safe<br>3 = Quite safe<br>2 = Quite unsafe<br>1 = Very unsafe<br>88 = No answer (Do not read)
Q9	Now we have some general questions about your life situation:<br>What is your gender?	1 = Man<br>2 = Woman<br>3 = Other

	Instruction: Do not read the answer options.	88 = No answer
Q10	Do you live alone?<br><br>*Instruction: Do not read the answer options.*	0 = No.<br>1 = Yes<br>88 = No response
Q11	How often can you get help and support from people nearby when you need it?	5 = Always<br>4 = Often<br>3 = Sometimes<br>2 = Rarely<br>1 = Never<br>88 = No answer (Do not read)
Q12	Do you have home care?<br><br>*Instruction: Do not read the answer options.*	0 = No.<br>1 = Yes<br>88 = No response
Q13	Are you able to get around indoors?	2 = Yes, independently<br>1 = Yes, with the help of aids or another person<br>0 = No.<br>88 = No answer (Do not read)
Q14	How would you describe your general health?	5 = Very good<br>4 = Pretty good<br>3 = So-So<br>2 = Pretty bad<br>1 = Very bad<br>88 = No answer (Do not read)
Q15	During the last 12 months, have you had any difficulty managing your finances?<br><br>*Instruction: Do not read the answer options.*	0 = No.<br>1 = Yes<br>88 = No response
Q16	Would you consider participating in an interview study where researchers from XXX University call you to ask further questions about unsafety in the home? If so, can I give them your name and phone number?<br><br>*Instruction: Do not read the answer options.*	0 = No.<br>1 = Yes<br>88 = No response
